# Systematic and quantitative analysis of stop codon readthrough in Rett syndrome nonsense mutations

**DOI:** 10.1007/s00109-024-02436-6

**Published:** 2024-03-02

**Authors:** Dennis Lebeda, Adrian Fierenz, Lina Werfel, Rina Rosin-Arbesfeld, Julia Hofhuis, Sven Thoms

**Affiliations:** 1 Department for Biochemistry and Molecular Medicine, Medical School EWL, Bielefeld University, Bielefeld, Germany; 2 Department of Child and Adolescent Health, University Medical Center Göttingen, Göttingen, Germany; 3 Department of Clinical Microbiology and Immunology, Faculty of Medicine, Tel Aviv University, Tel Aviv, Israel; 4 Present Address: Department of Pediatric Kidney, Liver and Metabolic Diseases, Hannover Medical School, Hannover, Germany

**Keywords:** Translational readthrough, Rare disease, Rett syndrome, *MeCP2*, Personalized medicine, Aminoglycoside

## Abstract

**Abstract:**

Rett syndrome (RTT) is a neurodevelopmental disorder resulting from genetic mutations in the *methyl CpG binding protein 2* (*MeCP2*) gene. Specifically, around 35% of RTT patients harbor premature termination codons (PTCs) within the *MeCP2* gene due to nonsense mutations. A promising therapeutic avenue for these individuals involves the use of aminoglycosides, which stimulate translational readthrough (TR) by causing stop codons to be interpreted as sense codons. However, the effectiveness of this treatment depends on several factors, including the type of stop codon and the surrounding nucleotides, collectively referred to as the stop codon context (SCC). Here, we develop a high-content reporter system to precisely measure TR efficiency at different SCCs, assess the recovery of the full-length *MeCP2* protein, and evaluate its subcellular localization. We have conducted a comprehensive investigation into the intricate relationship between SCC characteristics and TR induction, examining a total of 14 pathogenic *MeCP2* nonsense mutations with the aim to advance the prospects of personalized therapy for individuals with RTT. Our results demonstrate that TR induction can successfully restore full-length *MeCP2* protein, albeit to varying degrees, contingent upon the SCC and the specific position of the PTC within the *MeCP2* mRNA. TR induction can lead to the re-establishment of nuclear localization of *MeCP2*, indicating the potential restoration of protein functionality. In summary, our findings underscore the significance of SCC-specific approaches in the development of tailored therapies for RTT. By unraveling the relationship between SCC and TR therapy, we pave the way for personalized, individualized treatment strategies that hold promise for improving the lives of individuals affected by this debilitating neurodevelopmental disorder.

**Key messages:**

- The efficiency of readthrough induction at *MeCP2* premature termination codons strongly depends on the stop codon context.
- The position of the premature termination codon on the transcript influences the readthrough inducibility.
- A new high-content dual reporter assay facilitates the measurement and prediction of readthrough efficiency of specific nucleotide stop contexts.
- Readthrough induction results in the recovery of full-length *MeCP2* and its re-localization to the nucleus.
- *MeCP2* requires only one of its annotated nuclear localization signals.

**Supplementary Information:**

The online version contains supplementary material available at 10.1007/s00109-024-02436-6.

## Introduction

Rett syndrome (RTT) is a rare X-linked neurodevelopmental disorder that primarily affects females and shows a global incidence of approximately 1:10,000 [1]. Affected infants initially show normal postnatal development followed by an early disease onset between 6 and 18 months. Between the age of 1 year and 4 years, patients are presented with developmental arrest or loss of previously acquired skills. They often show a partial or complete loss of previously acquired speech, gait disorders, and loss of acquired hand motor functions, which is replaced by stereotypical hand movements. Abnormal social behavior may be present. Numerous facultative further symptoms, e.g., respiratory disturbances and cardiac arrhythmias, may occur [2, 3]. RTT symptoms usually worsen in terms of developmental regression, followed by a stage of symptom stabilization. In young adulthood, complications may occur including epilepsy or Parkinsonism-like symptoms. The likelihood of survival to the age of 20 varies between 78 and 95% [4]. In approximately 95% of patients, RTT is caused by one of over 900 recorded mutations in the *MeCP2* gene [5, 6].

*MeCP2* is a chromatin-associated transcription factor that can bind methylated CpG dinucleotides through a methyl-binding domain (MBD) to modulate gene transcription through a transcriptional repression domain (TRD). Two nuclear localization signals (NLS) have been reported that lead to the nuclear localization of *MeCP2* [7, 8]. Alternative initiation at exon 2 (*MeCP2*e2) and alternative splicing of exon 2 (*MeCP2*e1) lead to the expression of two protein isoforms with different N-termini [9, 10]. Both isoforms retain DNA-binding and methylating function but differ in their affinity for DNA and exhibit unique interacting protein partners [11]. MeCPe1 is the isoform critical for Rett syndrome [12].

*MeCP2* mutations mainly occur de novo and the eight most prevalent mutations are present in approximately 70% of all RTT patients [5, 13]. Remarkably, four of these eight mutations are nonsense mutations (p.R168X, p.R255X, p.R270X, p.R294X). About 35% of all RTT patients carry *MeCP2* nonsense mutations leading to premature termination codons (PTCs) which create truncated, nonfunctional protein products [13, 14]. A comparison between patients with C-terminal deletions shows that the disease severity correlates with the extent of the deletion [13]. Mouse models recapitulate many characteristics of the human phenotype and recent studies show that postnatal restoration of *MeCP2* reverses neurological symptoms even after the onset of RTT [15–17], indicating that, also in humans, the symptoms may be treatable. Currently, no curative therapies are available for RTT patients even though promising results were achieved using trofinetide in recent clinical trials [18, 19].

The aminoglycoside antibiotic geneticin (G418) efficiently binds to the bacterial 16S-rRNA inhibiting translation and protein synthesis. Due to a missing base pairing in the decoding center of the eukaryotic rRNA, geneticin binding to the ribosomes is hinged [20]. However, the interaction is sufficient to induce occasional disruptions of the ribosomal proofreading function which leads to an increased probability that a near-cognate tRNA (nc-tRNA) will bind to a stop codon, rather than a release factor. Hence, eukaryotic translational termination is suppressed and the elongation proceeds until the next in-frame stop codon is incorporated into the ribosomal A site which is referred to as translational readthrough (TR) [20, 21]. By inducing TR at PTCs with geneticin, the synthesis of full-length proteins instead of truncated variants can be achieved. This approach was used in animal and cell models of nonsense-derived rare diseases using various small molecules that induce TR [22–25].

The stop codon and its surrounding nucleotides are referred to as the stop codon context (SCC). The SCCs, especially the stop codon itself (nucleotides + 1 to + 3) and the + 4 nucleotide, are known to influence the probability of noninduced (basal) TR [26–28] and induced TR [25, 29, 30]. In general, UGA stop codons are most prone to TR and induction allows high TR efficiencies whereas UAA stop codons efficiently terminate the translation with low basal and inducible TR efficiencies.

TR induction of *MeCP2* nonsense mutations has been investigated in cellular models, patient-derived cells, and mouse models with a focus on the four most common nonsense mutations [17, 31–33]. To gain insights into TR induction of a broader patient-specific mutation spectrum, we analyzed the TR of 14 pathological *MeCP2* nonsense mutations to derive the context-dependent effect of geneticin treatment. Long-term application of aminoglycosides such as gentamycin is associated with oto- and nephrotoxicity. In the lab, geneticin gives important insights into stop codon suppression of various disease-related PTCs and can be regarded as a gold standard for TR inducibility [34]. Information about TR inducibility at SCCs can lay the foundation for patient-specific treatments using less toxic small molecules like ataluren [35] or aminoglycoside derivatives like the NB series [36].

**Fig. 1:**
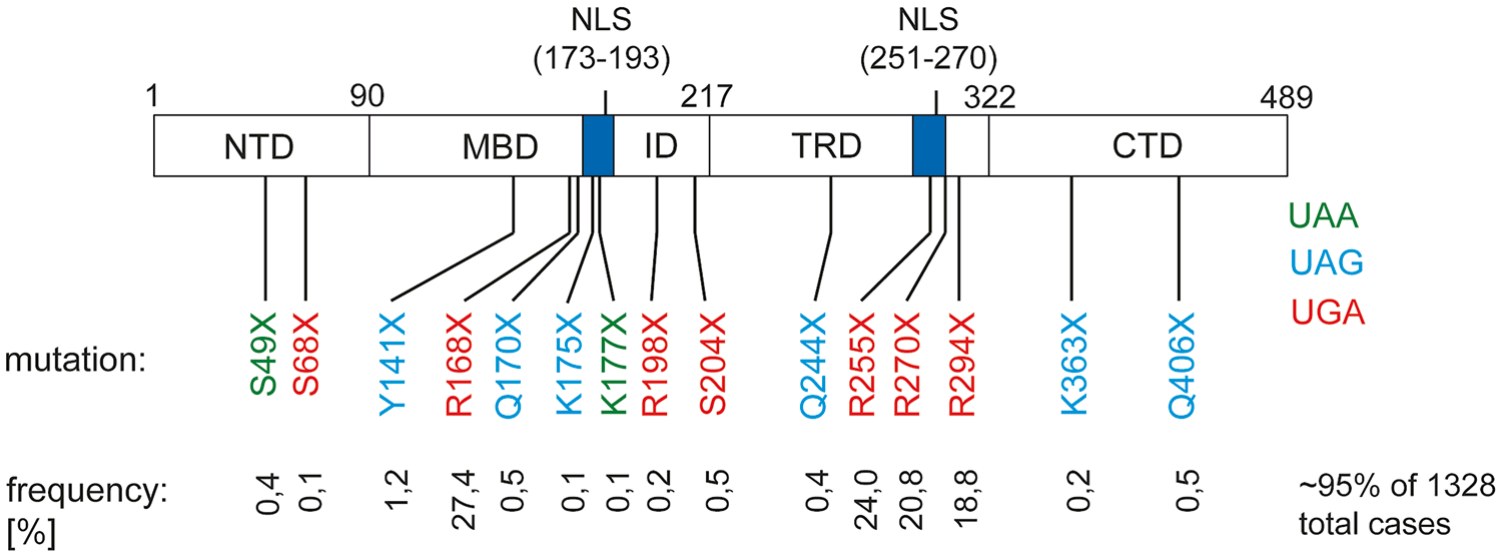
Pathological PTCs in the *MeCP2* coding sequence. NTD N-terminal domain, MBD methyl-binding domain, ID intervening domain, TRD transcriptional repression domain, CTD C-terminal domain; frequencies according to RettBASE

We analyzed the readthrough induction at stop codons and the following + 4 nucleotides specifically to see whether these positions are sufficient for the analysis of SCC readthrough in RTT. Our intention is to ultimately derive a correlation between the levels of translational readthrough, the SCC, and other genetic factors. A deeper understanding of the patient’s genetic variants will not only help improving (or, for that matter, enabling) readthrough therapy, but on the more experimental side, it will also reduce, refine, and replace (3R) animal experimentation, because it will enable us to devise the animal systems that are best suited for the advancement of readthrough therapy. In this study, we therefore quantified the SCC readthrough of 14 different disease-relevant nonsense mutations (Fig. 1) in an adapted high-content reporter system that allows the fast analysis of multiple conditions as well as the full-length protein restoration after TR induction. Further, the verification of correct subcellular recovered *MeCP2* localization confirmed our results from the SCC analysis. This highlights the advantage of SCC-based readthrough determination, e.g., by dual reporter analysis, in the context of personalized therapy of rare diseases.

## Material and methods

### Generation of the dual reporter and full-length constructs

SCCs of *MeCP2* PTCs (Suppl. Table 1) were cloned in the dual reporter system as described before [37]. The pcDNA3.1(+)GFP_MCS_RFP expression vector (PST1596) contains RFP in frame with a downstream GFP and was used as a 100% readthrough control as well as a vector for SCC insertion. The vector was opened by restriction with BspEI and BstEII. Oligonucleotides containing the corresponding SCCs consisted of 10 nucleotides upstream and downstream of each stop codon and BspEI as well as BstEII restriction site overhangs. Oligonucleotides (Suppl. Table 2) were annealed and ligated into the opened PST1596 vector fragment (Suppl. Fig. 2). The p3XFLAG-CMV 7.1 expression vector (PST1810) containing full-length *MeCP2* cDNA, and three nonsense variants (p.R168X, p.R255X, and p.R270X), were provided by Peter Huppke [31]. All other *MeCP2* mutations were introduced by site-directed mutagenesis.

### Cell culture

HeLa cells were cultured in Dulbecco’s Modified Eagle Medium (DMEM) supplemented with 1% glutamine, 1% penicillin/streptomycin, and 10% fetal calf serum (FCS) at 37 °C, 5% CO_2,_ and 90% humidity. 3 × 10^5^ cells/well were seeded in 6-well plates for cell lysis and immunoblotting, 1 × 10^5^ cells/well were seeded in 12-well plates for immunofluorescence, and 3 × 10^4^ cells/well were seeded in 96-well plates for the dual reporter assay. Cells were transfected by Effectene according to the manufacturer (Qiagen). 150 ng or 400 ng of plasmid DNA have been used for 96-well plates (dual reporter assay) or 6-well plates (full-length cDNA constructs), respectively. The transfection reagent was removed no later than 16 h after transfection. Cells were treated with a final concentration of 100 ng/µL geneticin (Carl Roth) for 24 h.

### High-content translational readthrough dual reporter assay

The principle of the dual reporter assay and TR quantification was described before [37]. Cells were washed with 150 µL phosphate-buffered saline (PBS), detached with 35 µL of 0.5% trypsin (Sigma) for seven minutes, resuspended in 165 µL DMEM (supplemented with 1% glutamine, 1% penicillin/streptomycin, and 10% FCS), and centrifuged for 5 min at 500xg. The supernatant was carefully removed and cells were thoroughly resuspended in 200 µL phenol red-free DMEM. 96-well plate flow cytometry (Guava EasyCyte 4th generation, Luminex) was performed using 488 nm and 532 nm lasers. Cell gating of HeLa cells was set from 23,000 to 75,000 forward scatter; and 12,500 to 67,500 side scatter signals. Gated cells with a higher RFP signal intensity than 700 or a higher GFP signal intensity than 100 were included in the following TR calculations. Background GFP signal from an RFP-expressing construct (PST1880) was subtracted from the GFP values of all wells. TR was then expressed as the ratio of GFP to RFP normalized to GFP to RFP of the 100% TR control vectors (PST1596). For each 96-well plate, PST1596 and untransfected HeLa cells were individually measured as triplicates. Gatings and calculations were performed using RStudio (R Core Team).

### Cell lysis and immunoblotting

Cells in 6-well plates were washed twice with 1 mL of ice-cold PBS, scraped off in another 1 mL of cold PBS, transferred into a new tube, and centrifuged for 5 min at 16,000xg. The supernatant was removed and cells were lysed by adding 70 µL RIPA buffer (150 mM NaCl, 50 mM Tris/HCl (pH 8.0), 5 mM EDTA (pH 8.0), 0.8% sodium deoxycholate, 1% Nonidet P-40, 0.1% SDS) with proteinase inhibitor Complete (Roche). After incubation on ice for 30 min, lysates were treated with 50 units DNaseI (Invitrogen) and 5 mM MgCl_2_, and incubated at 37 °C for 10 min for DNA digestion. Lysates were centrifuged for 20 min at 4 °C and 16,000xg to remove the insoluble fraction. Protein concentration in the supernatant was measured by a BCA assay (Interchim). 30 µg protein per sample was loaded onto an SDS-PAGE. For Western blotting, proteins were transferred to nitrocellulose membrane, which was blocked (5% milk powder in TBS-T (20mM Tris, 150mM NaCl, 0.1% Tween 20 detergent)) for 30 min at RT and probed overnight at 4 °C with the primary antibody (anti-Flag M2 (Merck F1804) and anti-GAPDH (Sigma G9545), 1:1000 each) and the secondary antibody (horseradish peroxidase-conjugated donkey anti-mouse IgG (715-035-150) or goat anti-rabbit IgG (111-035-003), Jackson Immuno-Research Laboratories, 1:10,000) for 1 h at RT. Reactive bands were revealed using Lumi-Light Plus Western blot substrate (Roche). Chemiluminescence was recorded using Chemostar Touch ECL & Fluorescence Imager (Intas Science Imaging). Densitometric analyses were performed in Fiji-ImageJ. Readthrough efficiency was calculated as the ratio of full-length *MeCP2* band intensity to the sum of full-length and truncated *MeCP2* band intensity. The relative *MeCP2* protein level was calculated as the ratio of PTC-containing *MeCP2* signal intensity to the intensity of wildtype *MeCP2* displayed on the same Western blot.

### Immunofluorescence and microscopy

For immunostaining, transfected cells on 18 mm coverslips (12-well plate) were washed with sterile PBS and fixed with 3.7% paraformaldehyde (Roth) for 10 min at RT. After three washing steps with PBS, cells were permeabilized with 0.5% TritonX-100 dissolved in PBS for 5 min at RT. Samples were blocked for 30 min at RT with 5% BSA in PBS. Primary antibody (anti-Flag M2 (Merck F1804), 1:500 in PBS and 1% BSA) incubation was done overnight at 4 °C followed by three washing steps with PBS. The secondary antibody (goat anti-mouse Alexa Fluor 647 (Invitrogen A32728), 1:1000) was incubated together with DAPI (0.1 μg/ml in PBS) and Alexa Fluor 488 phalloidin (Invitrogen, 1:400) in 1% BSA in PBS for 1 h at RT. Coverslips were mounted with ProLong™ Gold Antifade Mountant (Invitrogen) and left to dry overnight at RT. Images of the stained cells were acquired using the LSM900 confocal fluorescence microscope (Zeiss) using a 63X oil immersion objective. Image analysis, processing, and line plotting were done in Fiji-ImageJ.

### Statistical data analysis

The Lin’s CCC was calculated to evaluate the strength of agreement between the previously published TR assay and the high-content TR assay used in this work [38]. Further, a Bland-Altmann analysis was performed to determine the agreement of TR efficiencies obtained by both methods using GraphPad Prism’s built-in Bland-Altmann method comparison tool.

## Results

### A high-content flow cytometry assay for readthrough measurement

**Fig. 2:**
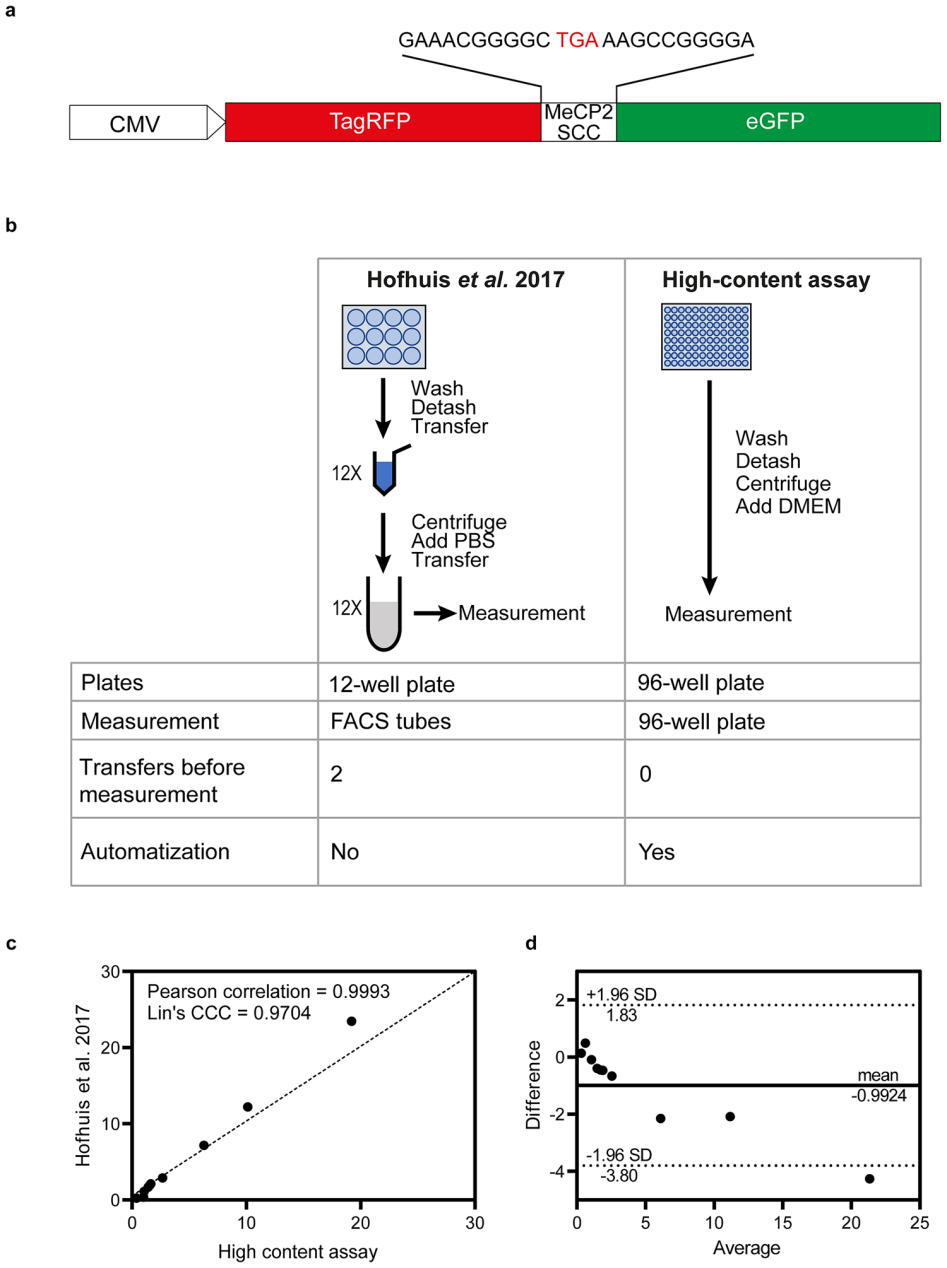
Comparison between the previously established dual reporter system for TR analysis and the novel high-content flow cytometry assay used in this work. **a** Dual reporter constructs for the analysis of TR. **b** The high-content assay allows the analysis of samples in a 96-well plate compared to a 12-well plate. The flow cytometer enables direct and automated measurement in the same 96-well cell culture plate which reduces time wasted by transferring each sample and material waste. **c** Pearson’s correlation and Lin’s concordance correlation coefficient (CCC) as well as **d** Bland-Altman analysis for TR efficiency measurements between the high-content TR assay and the previously established TR assay

To determine the influence of the SCC on the readthrough of various *MeCP2* PTCs, we used a dual reporter assay similar to a previously established system [27] in which reporter constructs code for a fusion protein consisting of an N-terminal TagRFP, the SCC region (-10 to +13 nucleotides), and a C-terminal eGFP (Fig. 2a). Cell-specific fluorescence was then measured by flow cytometry using reporter-transfected HeLa cells and TR was determined by the ratio of GFP to RFP fluorescence compared to a 100% TR control [25, 37].

Here, we improved the system and used a high-content flow cytometry approach to automatically measure samples in a 96-well plate. This approach allows fast and precise TR quantification of multiple SCCs at once, thereby reducing sample preparation time as well as material waste (Fig. 2b). Comparison of the previously established assay and the high-content TR assay was analyzed using Lin’s concordance correlation coefficient (CCC) and Bland-Altman analysis. Lin’s CCC compares two measurements of the same variable (each *MeCP2* SCC construct) and correlates both to a line of perfect fit (*y* = *x*) (Fig. 2c). The calculated Lin’s CCC of 0.9704 corresponds to a substantial concordance [39]. The Bland-Altman assay compares both assays and narrows down an acceptable range for the fluctuation of the differences. Comparing the high-content TR assay and the previously published TR assay, all measurement differences were in an acceptable range except for one measurement that exhibited a high TR efficiency, in agreement with the tendency of higher variance in high TR values in both assays (Fig. 2D). Further, three *MeCP2* SCCs bearing different stop codons show that the TR inducibility with geneticin is dependent on the stop codon in both systems, as expected (Suppl. Fig. 1). Hence, the high-content assay provides a faster, cheaper, and precise method for TR quantification of several samples at once. This system also allows the comparison between different TR-inducing compounds.

### Analysis of basal and stimulated readthrough of *MeCP2* SCCs by dual reporter assay

**Fig. 3:**
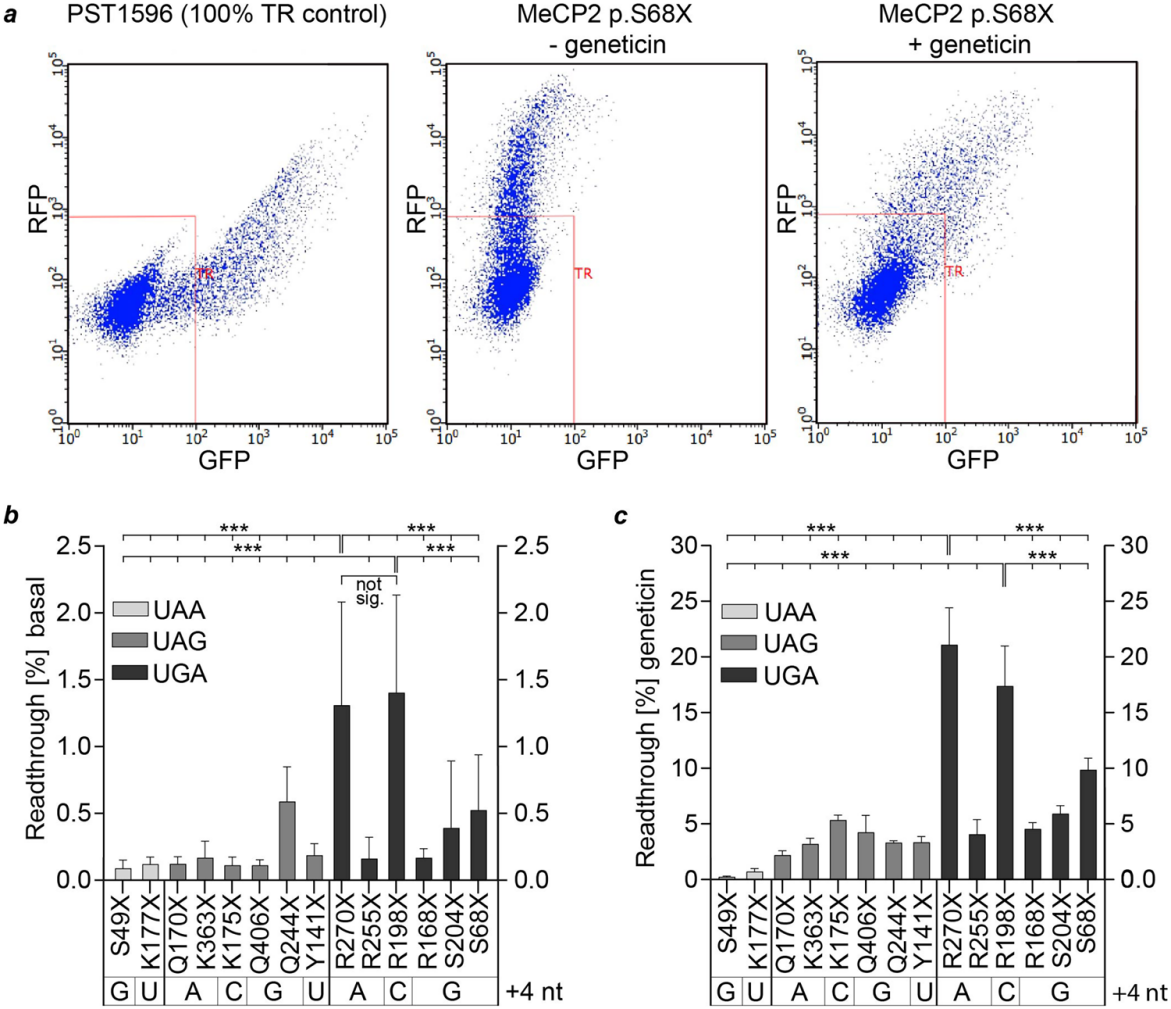
Basal and induced TR depend on the stop codon type and the context of the PTC. **a** Representative plot of individual HeLa cells after flow cytometry. Red squares gate untransfected HeLa cells with RFP signals < 700 and GFP signals < 100. Ten thousand events were recorded for each independent measurement. **b**, **c** Readthrough was estimated by the ratio of GFP to RFP which was normalized to the same ratio of a 100% TR control. Three replicates each were measured in three independent experiments. **b** Flow cytometric analysis shows that basal TR of each PTC is generally low (< 1%) except for p.R270X and p.R198X (*n* = 9). **c** TR efficiency after induction by geneticin at a concentration of 100 ng/µL depends on the SCC (*n* = 9, *n* = 6 for Q244X). Bar plots indicate mean; error bars indicate s.d. One-way ANOVA, with post hoc *P* values from Bonferroni’s test. *P < 0.033, **P < 0.002, and ***P < 0.001 vs R270X or R198X, respectively

In this study, we analyzed 14 pathogenic *MeCP2* nonsense mutations (Fig. 1) within their SCC in transfected HeLa cells using the high-content dual reporter assay (Fig. 3a). All tested mutations are associated with disease-causing pathogenicity that manifests in RTT or RTT-like phenotypes [6]. Comparison of the mean absolute RFP values does not show significant differences between the constructs (Suppl. Fig. 4). We therefore infer that mRNA and protein stabilities of the reporter constructs are not altered by the genetic variation.

Among the tested mutations, two contain UAA, six UAG, and further six the UGA stop codon. The stop codon itself as well as the surrounding context, especially a cytosine in position + 4, has previously been shown to have an impact on the TR efficacy [26, 40]. One of the analyzed *MeCP2* SCCs (p.R198X) contains the high-readthrough motif UGA C [26] but occurs at low frequencies according to RettBASE [6]. The four most frequent nonsense mutations p.R168X, p.R255X, p.R270X, and p.R294X have been extensively studied in the past [5, 31–33]. It has been shown that different mutation types are strongly associated with RTT severity and that mutations located further downstream may be less severe than mutations located further upstream [5, 17].

Measurement of unstimulated cells shows low basal TR efficiencies (~ 0.1%), except for p.Q244X (UAG G), p.R270X (UGA A), and p.R198X (UGA C) (Fig. 3b). p.R198X, the only UGA C context, showed the highest basal TR at 1.4%, as expected. With the exception of p.R255X and p.R168X, SCCs bearing a UAA or UAG exhibit a lower basal TR efficiency than UGA stop codons.

TR was stimulated by the treatment of transfected cells with geneticin (Fig. 3c). The treatment induced TR of all SCCs but the level was highly dependent on the corresponding SCC. Those bearing a UAA stop codon (p.S49X and p.K177X) showed readthrough inductions of less than 1%. UAG stop codons exhibit an intermediate inducible TR (approx. 3 to 4%) with the exception of p.K175X (5.35%) in which a cytosine is located in the + 4 position. TR induction of UGA stop codons varies between the different SCCs from ~ 4 to ~ 21%. Hence, TR of some UGA PTCs is as inducible as UAG PTCs indicating that the stop codon alone is not sufficient for TR predictions as further factors seem to tune the TR efficiency. Of note, the highest induction was found with p.R270X (21%) and p.R198X (17%). The latter also exhibited the highest basal TR confirming the impact of the SCC composition on both basal TR and the inducibility. p.R270X exhibits similar basal TR and an even higher inducible TR than p.R198X but lacks a cytosine at position + 4. This further indicates the importance of the nucleotides within the SCC in addition to the stop codon type and the downstream + 4 nucleotide. This finding is additionally supported by the comparison of p.R270X to p.R255X which bear the same stop codon and + 4 nucleotide but differ extremely in their basal and inducible TR efficiencies.

### Readthrough analysis of *MeCP2* PTCs in full-length cDNA

**Fig. 4:**
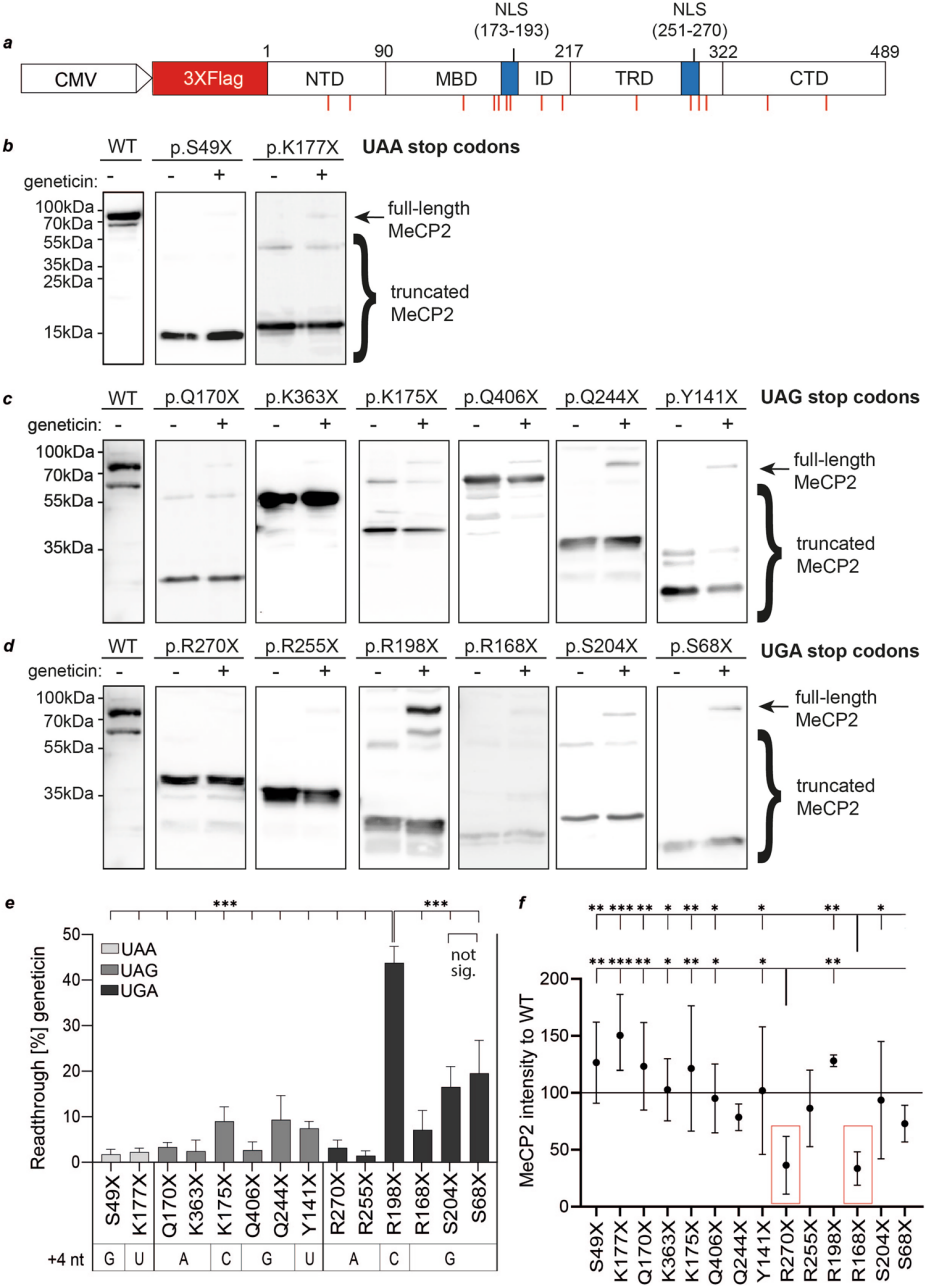
Recovery of full-length *MeCP2* by geneticin treatment. **a** N-terminal 3XFlag-tagged full-length *MeCP2* bearing individual pathological PTCs (red lines). **b**–**d** Western blots of *MeCP2* mutants using anti-Flag antibody after TR induction sorted by their PTC identity. After transfection, cells were treated for 24 h with 100 ng/µL geneticin. **e** Quantification of *MeCP2* PTC readthrough without or with geneticin treatment, respectively. Readthrough corresponds to the full-length *MeCP2* signal intensity normalized to the intensity of full-length plus truncated *MeCP2*. Bar plots indicate mean; error bars indicate s.d. One-way ANOVA, with post hoc *P* values from Bonferroni’s test. *P < 0.033, **P < 0.002, and ***P < 0.001 vs. R198X. **f** Relative level of PTC-containing *MeCP2* proteins. The protein level corresponds to the PTC-containing *MeCP2* signal intensity normalized to the intensity of wildtype *MeCP2* displayed on the same Western blot. Dots indicate mean, error bars indicate s.d. One-way ANOVA. *P < 0.05, **P < 0.01, and ***P < 0.005 vs. R.168X or R270X, respectively. *n* = 3 to 4 investigated, independent cell lysates

We next investigated readthrough in PTC variants of the full-length mRNA of *MeCP2*. cDNA tagged by N-terminal 3XFlag was mutated to obtain the 14 *MeCP2* nonsense mutations (Fig. 4a, Suppl. Figs. 2 and 3). HeLa cells were transfected and expression of full-length or truncated 3XFlag-*MeCP2* isoforms was analyzed by Western blotting using anti-Flag antibodies. Transfected HeLa cells were untreated or treated with geneticin for TR induction for 24 h before lysis.

All examined *MeCP2* nonsense mutations expressed a truncated *MeCP2* variant but lacked the expression of full-length *MeCP2* under untreated conditions showing that TR at PTCs did not occur (Fig. 4b–d). Only the *MeCP2* p.R198X mutation (Fig. 4c) showed a weak band at the position of wildtype *MeCP2*, indicating a high basal TR for this specific mutation which is consistent with the findings of the dual reporter assay. After geneticin treatment, full-length *MeCP2* was detectable for each mutation at greatly different intensities, which is also consistent with our previous results.

Next, the readthrough level was quantified as the ratio of recovered full-length *MeCP2* intensity to the intensity of total *MeCP2* (Fig. 4e). In agreement with the dual reporter experiment, the UAA stop codon displayed poor inducibility (S49X: 1.62%; K177X: 2.1%). Further, TR induction of most *MeCP2* UAG stop codons was usually intermediate between UAA and UGA stop codons with exception of the weak full-length recovery in p.K363X (2.3%) and p.Q406X (2.5%) which were as low as both UAA nonsense mutations. Interestingly, full-length recovery of the three most downstream nonsense mutations (p.R270X, p.K363X, p.Q406X) was weak although the corresponding SCCs exhibited intermediate to good inducibilities in the dual reporter assay, indicating a critical positional effect of PTCs on TR. p.R255X performed poorly (Fig. 4d, e), despite the UGA stop codon, like in the dual reporter assay before. The *MeCP2* p.R270X protein showed significantly reduced intensity ratio to wildtype *MeCP2* (Fig. 4f) which might explain the discrepancy in TR induction between the dual reporter construct and the full-length construct.

Overall, the *MeCP2* full-length recovery by geneticin treatment is efficient with restoration of up to ~ 40% and highly consistent with the results that were obtained in the dual reporter assay. These results support the critical influence of the SCC on TR and demonstrate the reliability of our dual reporter system. Such assays could prove useful to study multiple patient-specific nonsense mutations and multiple TR-inducing drugs in a high-content system.

### Recovery of subcellular localization of *MeCP2*

**Fig. 5:**
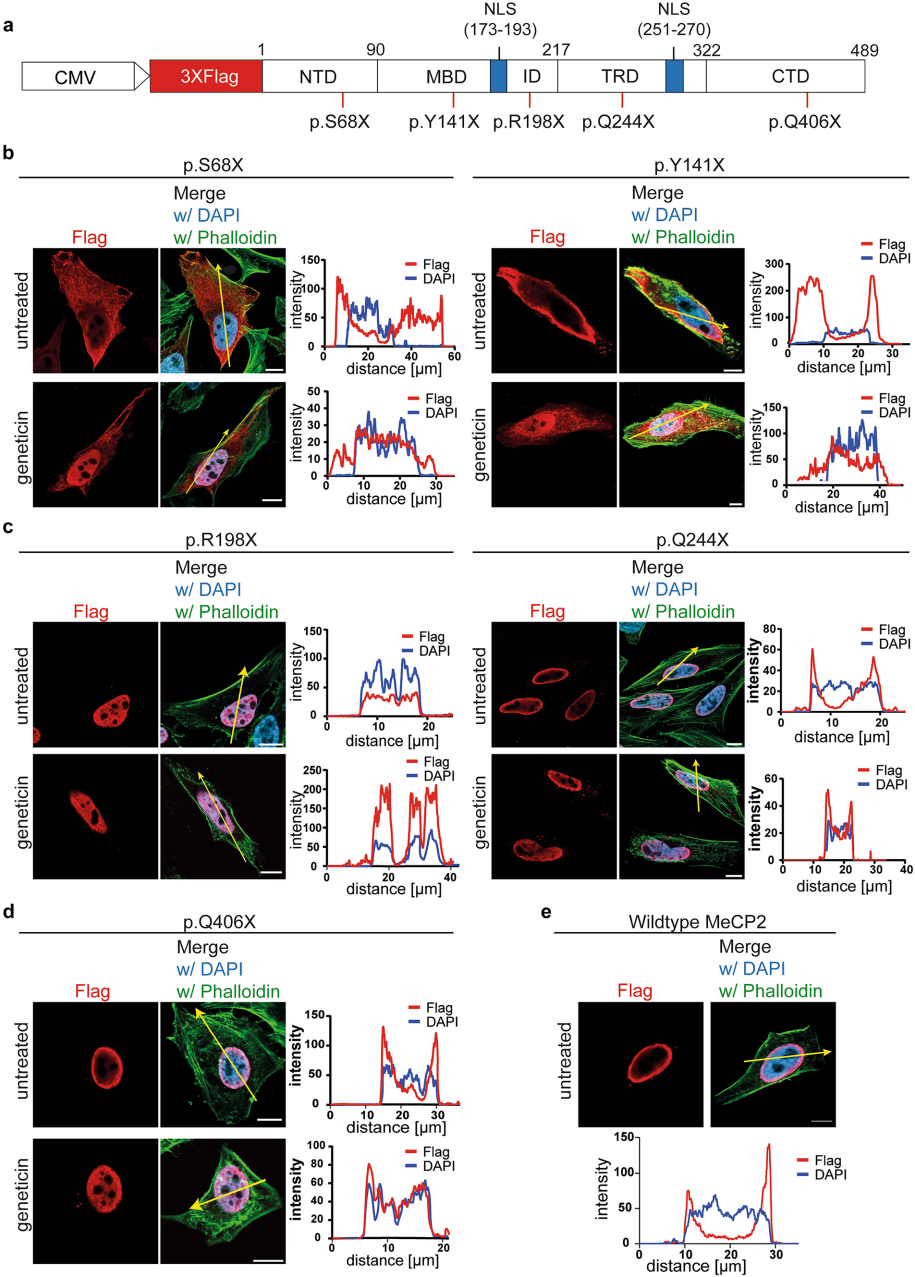
Subcellular localization of wildtype and PTC-bearing *MeCP2* upon geneticin treatment. **a** Constructs used for subcellular localization experiments. N-terminally 3XFlag-tagged *MeCP2* constructs bear no mutation or one of the following: p.S68X, p.Y141X, p.R198X, p.Q244X, and p.Q406X (red lines). Blue bars indicate the NLS positions. **b**–**e** *MeCP2* localization of transfected HeLa cells was investigated by immunostaining and fluorescence microscopy. Cells were untreated or treated with 100 ng/µL geneticin for 24 h before fixing. A histogram shows Flag (red) and DAPI (blue) signals along the yellow drawn arrow. **b** *MeCP2* p.S68X and *MeCP2* p.Y141X lack both NLSs shifting the nuclear to a cytoplasmic localization. Treatment with geneticin partially recovers the nuclear localization of *MeCP2*. **c** *MeCP2* p.R198X and *MeCP2* p.Q244X bear PTCs between both NLSs and localize to the nucleus without and with geneticin treatment, respectively. **d** *MeCP2* p.Q406X contains a PTC downstream of both NLSs and also localizes to the nucleus without and with geneticin treatment, respectively. Scale bar 10 µm. **e** Wildtype *MeCP2* completely localizes to the nucleus. *N* > 10 transfected cells in two independent immunofluorescence measurements

The *MeCP2* protein includes two nuclear localization signal (NLS) domains [7, 41, 42] at amino acids 173–193 and 251–270, respectively (Fig. 5a). To investigate the effect of readthrough stimulation on nuclear localization, we transfected cells with wildtype or one of five nonsense *MeCP2* constructs. Two PTC mutations are located upstream of both NLSs (p.S68X and p.Y141X, Fig. 5b), two are located between the NLSs (p.R198X and p.Q244X, Fig. 5c), and one is located downstream of both NLSs (p.Q406X, Fig. 5d). Cells were treated with geneticin and imaged after immunofluorescent cell staining using anti-Flag antibodies (Fig. 5b–e).

In the *MeCP2* p.S68X and p.Y141X variants, the nuclear localization of *MeCP2* is disrupted as the PTCs lead to the expression of a truncated *MeCP2* protein lacking both NLSs. Thus, the protein was detected in the cytosol (Fig. 5b). TR induction, on the other hand, partially restores the nuclear localization for both *MeCP2* variants. In accordance with the immunoblotting results, full-length protein recovery by induction occurs only partially and is highly dependent on the SCC so that truncated *MeCP2* was also detected in the cytosol. Recovery of nuclear localization in the case of *MeCP2* p.S68X (UGA G) was more efficient than in *MeCP2* p.Y141X (UAG U), presumably because the p.S68X SCC is more prone to readthrough induction as demonstrated in dual reporter and Western blot experiments.

*MeCP2* p.R198X and p.Q244X contain PTCs downstream of one NLS (Fig. 5c). Even without TR induction, both *MeCP2* variants were entirely localized to the nucleus similarly to wildtype *MeCP2* (Fig. 5e). This shows that the one NLS is sufficient for nuclear import and recovery of the downstream NLS is not necessary for correct localization. Consequently, *MeCP2* p.Q406X bearing both NLS domains also exhibits nuclear localization without readthrough induction (Fig. 5d).

## Discussion

In the current study, we show that a high-content dual reporter assay is useful for measuring the TR inducibility of specific PTC nucleotide contexts which can be verified by full-length recovery of *MeCP2* except for C-terminal positioned PTCs. This allows patient-specific TR investigation on the one hand and the use of various TR-inducing drug efficacies on the other hand. The latter is of importance to find less toxic drugs than aminoglycosides which induce oto- and nephrotoxicity at current therapeutic doses [43].

Targeting nonsense-mutated transcripts by readthrough induction for the recovery of full-length proteins can provide a useful option for the therapy of rare diseases [44]. Here, we created reporter constructs to characterize the SCC dependency on basal and inducible TR of 14 pathogenic *MeCP2* nonsense mutations. We demonstrated relevant differences in basal and induced TR efficiencies between all investigated *MeCP2* pathogenic variants. In general, basal TR is low. However, two variants, p.R270X and p.R198X, exhibit basal readthrough of more than 1%. Both bear UGA stop codons which are generally most prone to TR [25–27]. The *MeCP2* p.R198X variant further contains a cytosine in the + 4 SCC position which was previously shown to additionally increase the TR efficiency [26, 45]. Further, p.R198X was the only variant that expressed the full-length *MeCP2* isoform confirming the high basal TR of the UGA C context. Due to the low frequency of the p.R198X variant, nothing is known about an alleviated disease severity that might be a result of an enhanced basal TR. The better-characterized p.R270X mutation, however, is associated with a severe disease phenotype that is comparable to the nonsense mutations p.R168X, p.R255X, splice site mutations, deletions, and insertions [5]. All other variants do not show detectable full-length *MeCP2* isoforms but truncated variants which confirm the generally low basal TR.

TR induction with geneticin leads to the recovery of full-length *MeCP2* for each nonsense mutation variant. However, a strong dependency on the stop codon type and the context was observed. UAA PTCs show a weak recovery of full-length *MeCP2* compared to the other stop codons which is also observed in other diseases [25, 26]. Most UAG-containing variants show low to moderate full-length recoveries between UAA and UGA levels. UGA stop codons display the highest recovered full-length intensities. The UGA C p.R198X variant exhibits the highest rescued full-length intensity of all investigated variants underlining the high impact of the stop codon and + 4 nucleotide on TR.

Even though full-length recovery largely corresponds to the dual reporter readthrough levels, the full-length recovery of p.K363X, p.Q406X, and p.R270X variants turned out to be remarkably low in comparison to the corresponding dual reporter measurement, indicating the existence of factors that directly influence TR efficiency other than the SCC. The position of PTCs on the mRNA and its proximity to the poly(A) tail negatively influence TR efficiency [46]. Interestingly, the PTCs of p.K363X and p.Q406X are the furthest 3′ on the *MeCP2* transcript which might further indicate a critical role of the PTC position for TR, an effect that is not mirrored in the dual reporter assay with all SCCs located at the same relative position. The same effect can be seen for p.R270X, which displays the highest induced TR in the dual reporter assay but a remarkably low full-length recovery similar to UAA stop codons. The *MeCP2* p.R270X and p.R168X protein variants are significantly less stable than most other investigated protein variants. A cellular control mechanism that degrades mRNA molecules containing PTCs is the nonsense-mediated decay (NMD) [47]. The canonical recognition by the NMD mechanism relies on splicing of introns that are not present in our cDNA constructs. Therefore, the decreased protein expression cannot be explained by canonical NMD—the mRNA and/or the protein may be destabilized by a noncanonical form of NMD or the unfavorable protein structures or physicochemical properties. This instability is an explanation for the discrepancy of the p.R270X TR when dual reporter and unstable full-length constructs are compared. Interestingly, the low stability of the p.R270X gene product might explain the severe RTT phenotype in these patients [5].

All PTC variants located upstream of cDNA position c.808 (mutation results in the *MeCP2* p.R270X variant) show induction levels that are similar between dual reporter experiments and full-length recovery of *MeCP2*. The dual reporter assay provides a fast high-content quantification of TR that detects even low levels of basal TR [37]. Therefore, this system provides a powerful tool to investigate the inducibility of patient-specific PTC readthrough and of less toxic readthrough inducers, such as those that are currently being tested in disease-related studies [22, 24, 48]. Our results emphasize the importance of validating the dual reporter measurements by the use of the corresponding native full-length sequence since factors other than the SCC such as the position of the PTC on the mRNA or the protein stability might affect the level of TR.

We show that the investigation of PTCs and the + 4 nucleotide solely is not sufficient to derive a general PTC readthrough prediction. Although the UGA C variant p.R198X exhibits the highest basal and induced readthrough and most variants show the known stop codon–dependent inducibility of UGA > UAG > UAA, some PTC variants could be stimulated to unexpected readthrough levels. For example, the variants p.R168X, p.S204X, and p.S68X contain the same UGA G region but TR induction varies twofold in both, dual reporter and full-length recovery experiments. Further, the TR of the p.R255X UGA PTC is not inducible to levels that are distinguishable from UAG variants. The recovered protein level is even similar to the low recoveries of UAA PTCs. The weak induction of the p.R255X PTC using geneticin has also been shown in a recent study [17]. The reason for these outliers may be due to additional positions in the SCC that may have critical effects on TR efficiency as shown previously [25, 26, 28, 29, 49]. Therefore, future predictions of patient-specific PTC readthrough induction should include analyses of a broader SCC spectrum and analysis of TR induction using a set of TR-inducing drugs. As concluded before, the location of a PTC on the mRNA may be critical for the TR inducibility and should be considered for future patient-specific therapeutic TR studies.

*MeCP2* requires an NLS domain for transport into the nucleus and transcription factor activity [42, 50]. Our data indicate that the downstream NLS at amino acids 251–270 is not essential and the upstream NLS is sufficient for nuclear localization of *MeCP2* [8]. Another study has also demonstrated the apparent absence of function for the second NLS [51]. We demonstrated that PTC-containing *MeCP2* variants are partially re-localized to the nucleus after TR induction. The insertion of unspecific amino acids through near-cognate tRNAs at the PTC positions does not disturb the downstream nuclear localization function. Assessment of fully functional protein recovery would require further biochemical assays but our data indicate that readthrough induction is a promising approach to recover the functional full-length protein.

Taken together, we demonstrate that readthrough stimulation of *MeCP2* PTCs can reach a wide range of induction levels between 1 and 40% in a fashion that is highly dependent on the SCCs. Although the full-length recovery rate for some PTC-containing *MeCP2* variants appears low, the rescue of a small percentage might be sufficient to alleviate RTT disease phenotypes. Recovered full-length *MeCP2* protein also exhibits nuclear localization suggesting functionality. Future diagnostic approaches should make use of similar high-throughput analysis methods and investigate the options of TR induction in a patient-specific manner. Of note, this study covers the impact of patient-specific SCCs alone on the level of TR and lays the foundation for the selection of a set of suitable SCCs that would warrant the development of cellular or animal models to investigate neuronal development, synapse formation, and learning. By enabling readthrough therapy, we may be able to address not only Rett syndrome but also other genetic disorders at the molecular level. Concurrently, in line with our commitment to the principles of 3R (reduce, refine, replace), the approach used in this study minimizes the use of animal experiments and refines experimental protocols for enhanced animal welfare, because it allows to specify the most rewarding genetic constellation for TR therapy.

### Supplementary Information

Below is the link to the electronic supplementary material.

Supplementary file1 (PDF 1338 KB)

## Data Availability

The data that support the findings of this study are detailed in the supplementary material. Further data are available from the corresponding author upon reasonable request.
