# Supplementary material for: Systematic and quantitative analysis of stop codon readthrough in Rett syndrome nonsense mutations
Source: J Mol Med (Berl). 2024 Mar 2;102(5):641–53. doi: 10.1007/s00109-024-02436-6 (PMC11055764; doi:10.1007/s00109-024-02436-6)
Supplement: Supplementary file 1 — Supplementary file1 (PDF 1338 KB) [file 109_2024_2436_MOESM1_ESM.pdf]

Systematic and quantitative analysis of stop codon readthrough in Rett syndrome nonsense mutations

Dennis Lebeda, Adrian Fierenz, Lina Werfel, Rina Rosin-Arbesfeld, Julia Hofhuis, Sven Thoms

Supplemental Figures

Supplementary Table 1: Dual reporter plasmids generated in this study, the corresponding plasmid numbers (PST), and used oligonucleotides. Pathogenic mutation leading to a PTC is marked in red.

Plasmid PST	Specification	Oligonucleotide 1		Oligonucleotide 2	
		OST	Sequence	OST	Sequence
1596	STOP absent				
1600	p.R198X	1419	GTCACCGCGGCACCACGTGA CCCAAGGCGGT	1420	CCGGACCGCCTTGGGTCACGT GGTGCCGCG
1601	p.S49X	1421	GTCACCGCGTGCAGCCATAA GCCCACCACTT	1422	CCGGAAGTGGTGGGCTTATGG CTGCACGCG
1602	p.Q406X	1423	GTCACCGCCCTGAGCCCTAG GACTTGAGCAT	1424	CCGGATGCTCAAGTCCTAGGG CTCAGGGCG
1603	p.R294X	1425	GTCACCGGTCTTCTATCTGA TCTGTGCAGGT	1426	CCGGACCTGCACAGATCAGAT AGAAGACCG
1739	p.R168X	3014	GTCACCGCCCCTCCCGGTGA GAGCAGAAACT	3015	CCGGAGTTTCTGCTCTCACCG GGAGGGGCG
1740	p.R270X	1579	GTCACCGGAAACGGGGCTGA AAGCCGGGGAT	1580	CCGGATCCCCGGCTTTCAGCC CCGTTTCCG
1741	p.Y141X	3085	GTCACCGGTTGATTGCGTAG TTCGAAAAGGT	3086	CCGGACCTTTTCGAACTACGC AATCAACCG
1742	p.Q170X	1583	GTCACCGCCGGCGAGAGTAG AAACCACCTAT	1584	CCGGATAGGTGGTTTCTACTC TCGCCGGCG
1743	p.R255X	1585	GTCACCGCGGCAGGAAGTGA AAAGCTGAGGT	1586	CCGGACCTCAGCTTTTCACTT CCTGCCGCG
1744	p.S204X	1587	GTCACCGGGCGGCCACGTGA GAGGGTGTGCT	1588	CCGGAGCACACCCTCTCACGT GGCCGCCCG
1745	p.Q244X	1589	GTCACCGCACATCCACCTAG GTCATGGTGAT	1590	CCGGATCACCATGACCTAGGT GGATGTGCG
1746	p.K175X	3087	GTCACCGACCACCTAAGTAG CCCAAATCTCT	3088	CCGGAGAGATTTGGGCTACTT AGGTGGTCG
1747	p.S68X	1593	GTCACCGATCAGAAGGGTGA GGCTCCGCCCT	1594	CCGGAGGGCGGAGCCTCACCC TTCTGATCG
1748	p.K177X	1595	GTCACCGTAAGAAGCCCTAA TCTCCCAAAGT	1596	CCGGACTTTGGGAGATTAGGG CTTCTTACG
1749	p.K363X	3089	GTCACCGCTCACCCCCCTAG AAGGAGCACCT	3090	CCGGAGGTGCTCCTTCTAGGG GGGTGAGCG

Supplementary Table 2: Full-length MeCP2 plasmids generated in this study, the corresponding plasmid numbers (PST), and used oligonucleotides for mutagenesis. Mutated nucleotide leading to a PTC is marked in red.

Plasmid PST	Specification	Oligonucleotide 1		Oligonucleotide 2	
		OST	Sequence	OST	Sequence
1810	Wildtype MeCP2 cDNA				
1814	c.423C>G, p.Y141X	1619	CTCTAAAGTGGAGTTGATTGCGTAGTTCGAAAAGGTAGGCGACAC	1620	GTGTCGCCTACCTTTTCGAACTACGCAATCAACTCCACTTTAGAG
1815	c.508C>T, p.Q170X	1621	GCCCCTCCCGGCGAGAGTAGAAACCACCTAAGAAGC	1622	GCTTCTTAGGTGGTTTCTACTCTCGCCGGGAGGGGC
1816	c.611C>G, p.S204X	1623	GACCCAAGGCGGCCACGTGAGAGGGTGTGCAGGTG	1624	CACCTGCACACCCTCTCACGTGGCCGCCTTGGGTC
1817	c.730C>T, p.Q244X	1625	GGGCCACCACATCCACCTAGGTCATGGTGATCAAACGC	1626	GCGTTTGATCACCATGACCTAGGTGGATGTGGTGGCCC
1818	c.523A>T, p.K175X	1627	GAGAGCAGAAACCACCTAAGTAGCCCAAATCTCCCAAAGCTCC	1628	GGAGCTTTGGGAGATTTGGGCTACTTAGGTGGTTTCTGCTCTC
1819	c.203C>G, p.S68X	1629	GCAGAGACATCAGAAGGGTGAGGCTCCGCCCCGGCTG	1630	CAGCCGGGGCGGAGCCTCACCCTTCTGATGTCTCTGC
1820	c.529A>T, p.K177X	1631	GCAGAAACCACCTAAGAAGCCCTAATCTCCCAAAGCTCCAGG	1632	CCTGGAGCTTTGGGAGATTAGGGCTTCTTAGGTGGTTTCTGC
1821	c.1087A>T, p.K363X	1633	GCGCCTCCTCACCCCCCTAGAAGGAGCACCACCACC	1634	GGTGGTGGTGCTCCTTCTAGGGGGGTGAGGAGGCGC
1847	c.146C>A, p.S49X	1710	GCCCGTGCAGCCATAAGCCCACCACTCTGC	1711	GCAGAGTGGTGGGCTTATGGCTGCACGGGC
1848	c.1216C>T, p.Q406X	1712	GCCCCCCTGAGCCCTAGGACTTGAGCAGCAG	1713	CTGCTGCTCAAGTCCTAGGGCTCAGGGGGGC
1849	c.592A>T, p.R198X	1714	GGAGCGGCACCACGTGACCCAAGGCGGCC	1715	GGCCGCCTTGGGTCACGTGGTGCCGCTCC

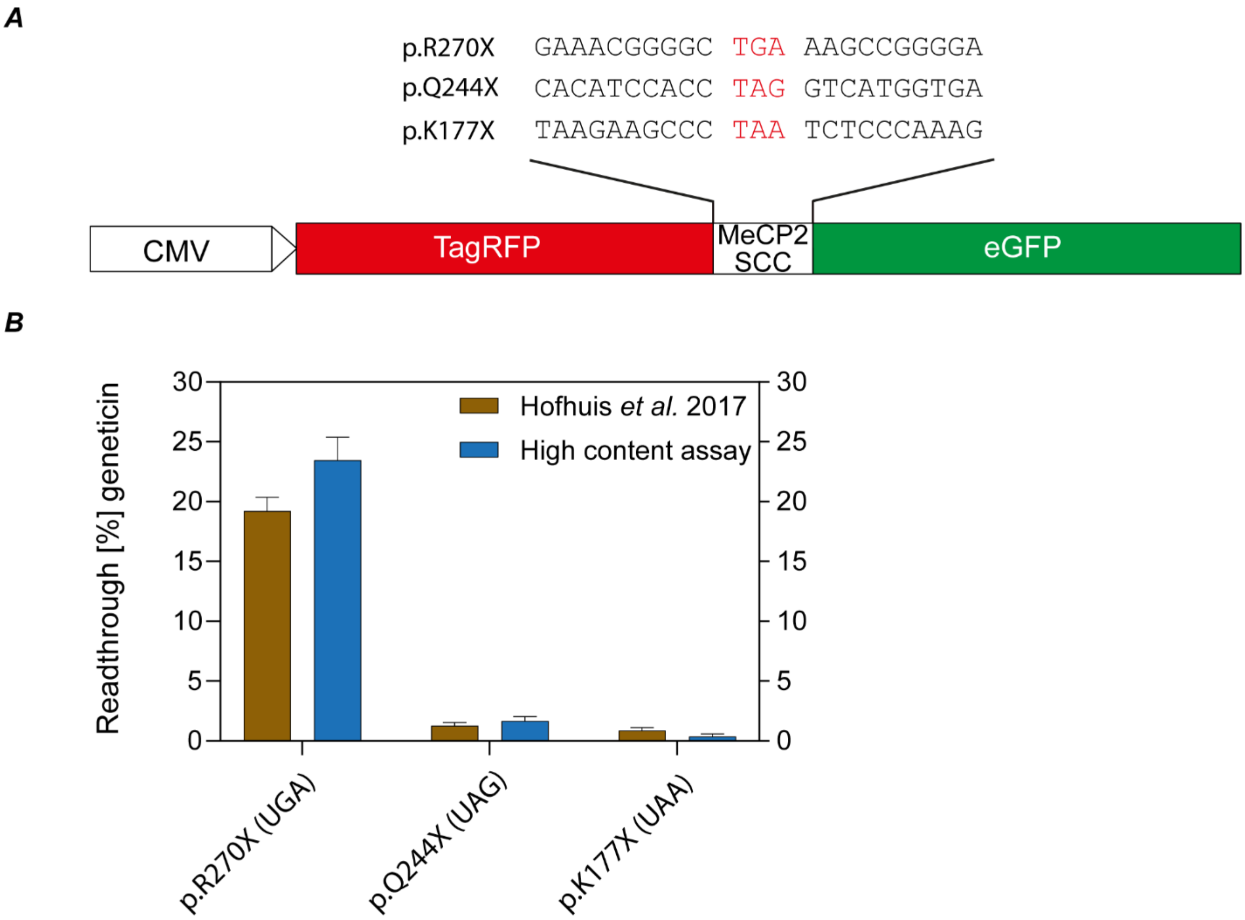

Supplementary Figure 1: Assessment of high-content dual reporter TR assay. **(A)** Three reporter constructs with different stop codons were used for the comparison between both assays. **(B)** TR measurement of the reporters in both assays using transfected HeLa cells

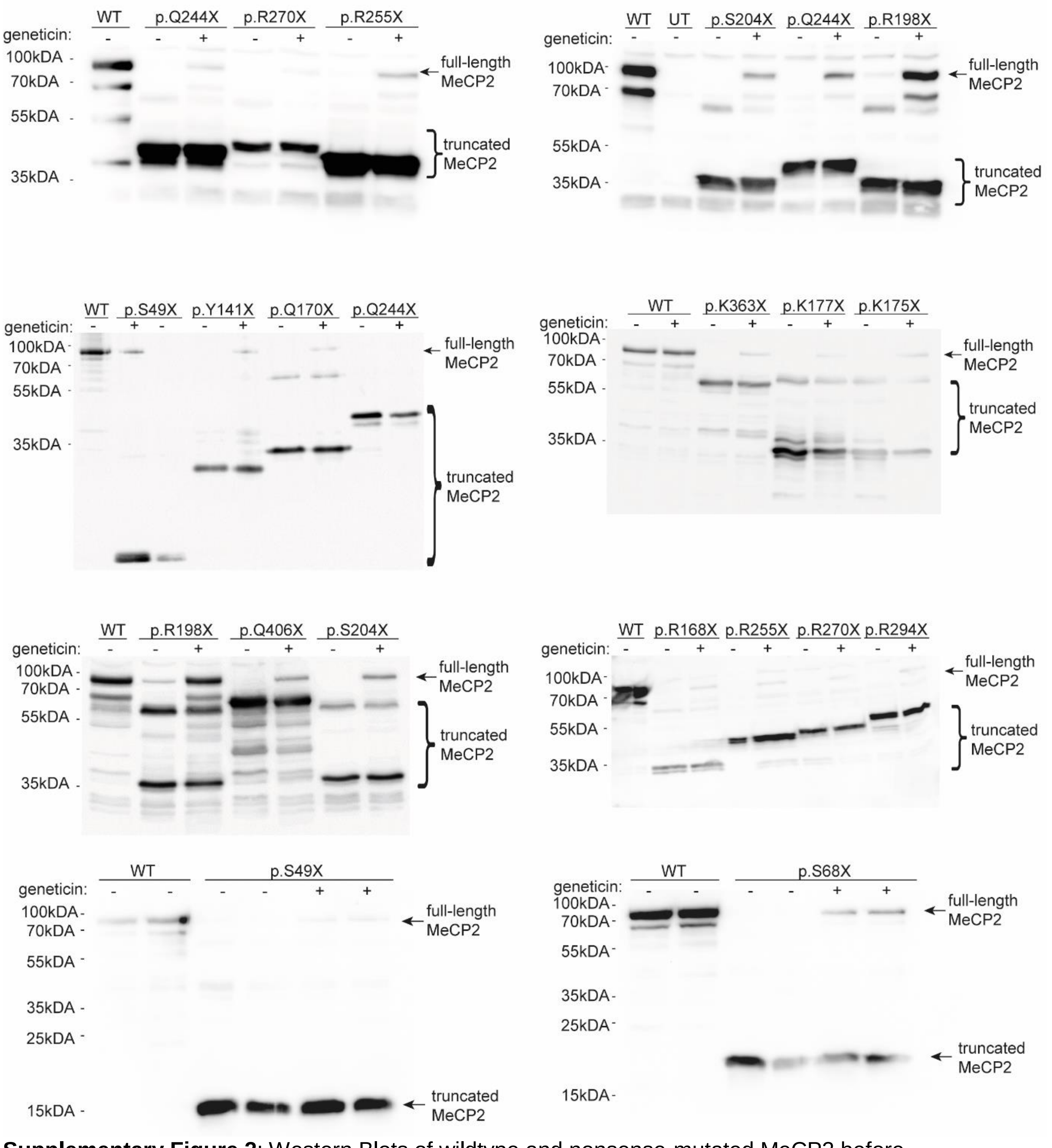

Supplementary Figure 2: Western Blots of wildtype and nonsense-mutated MeCP2 before and after geneticin treatment.

WB1

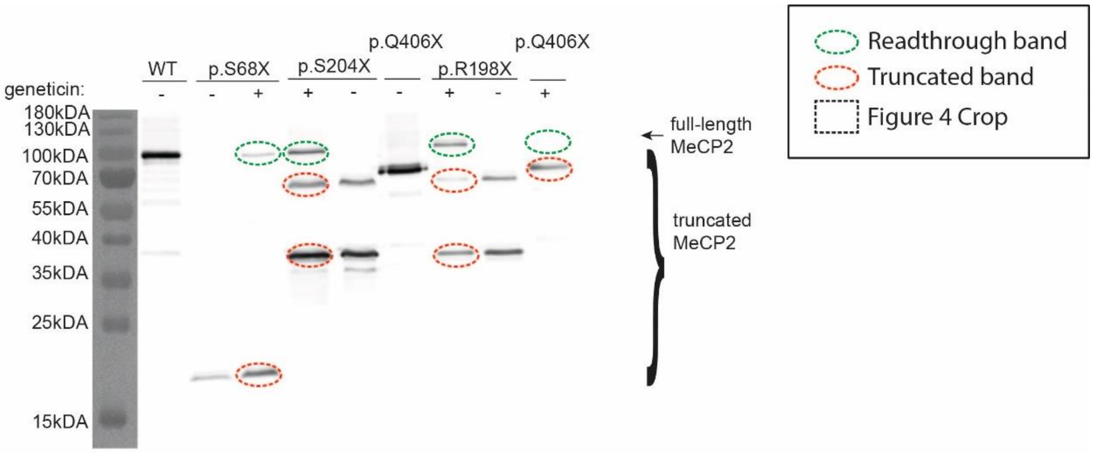

WB2

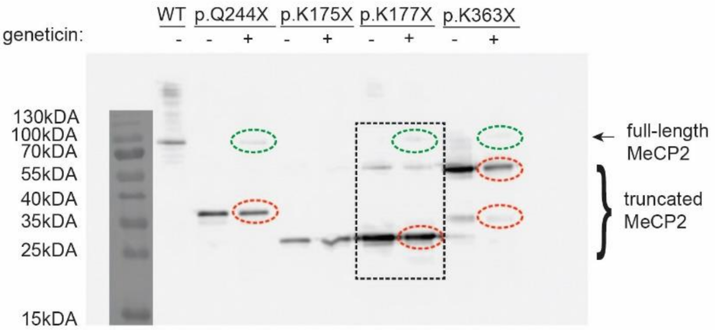

used in Figure 4

WB3

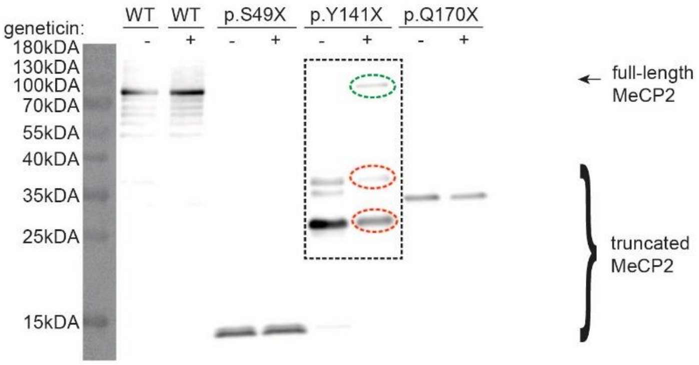

Used in Figure 4

WB4

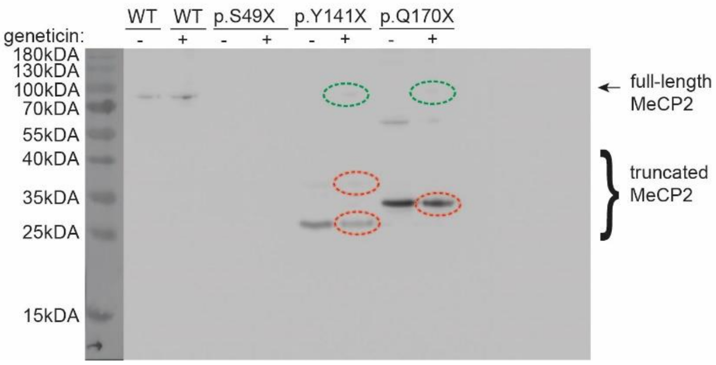

WB5

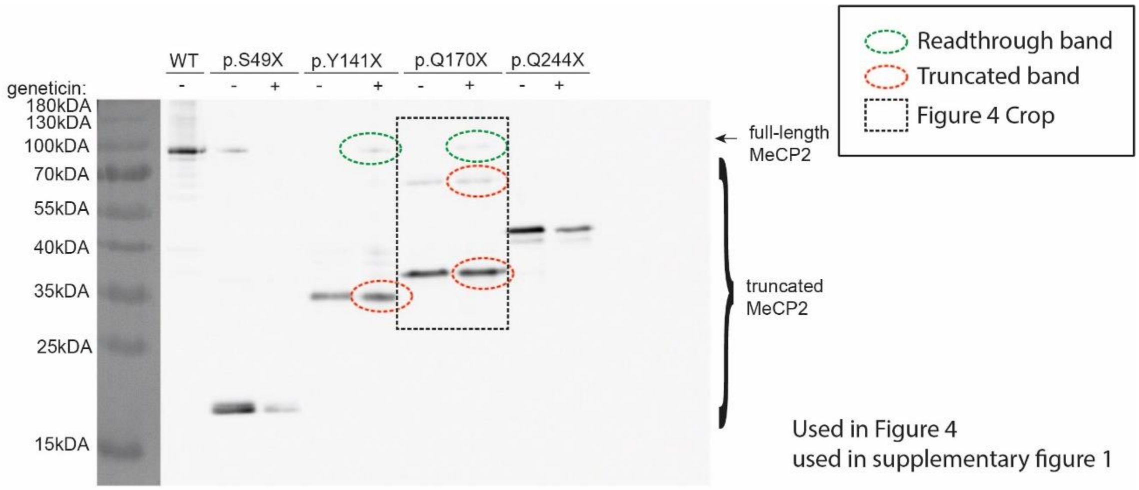

Used in Figure 4
used in supplementary figure 1

WB6

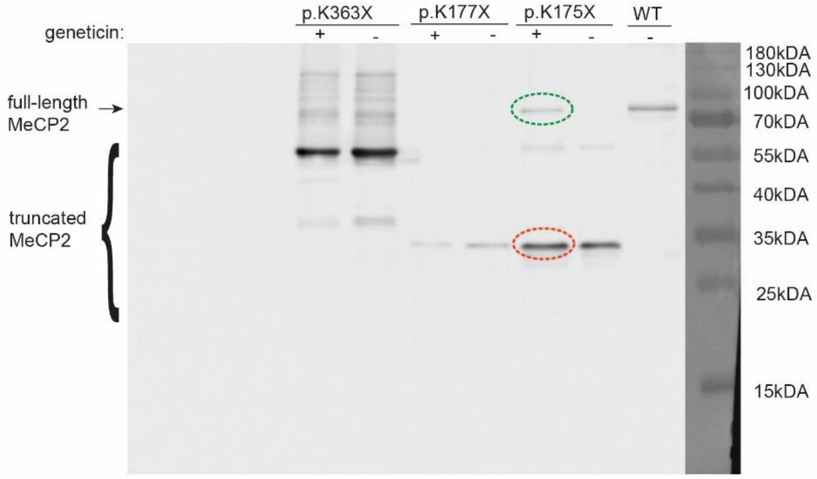

WB7

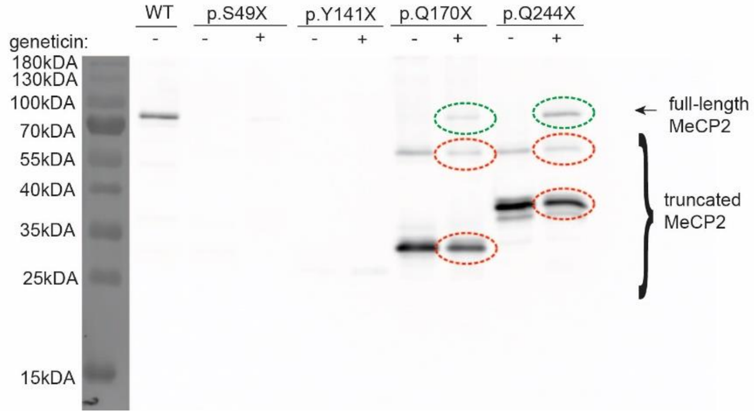

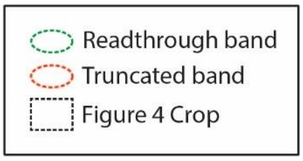

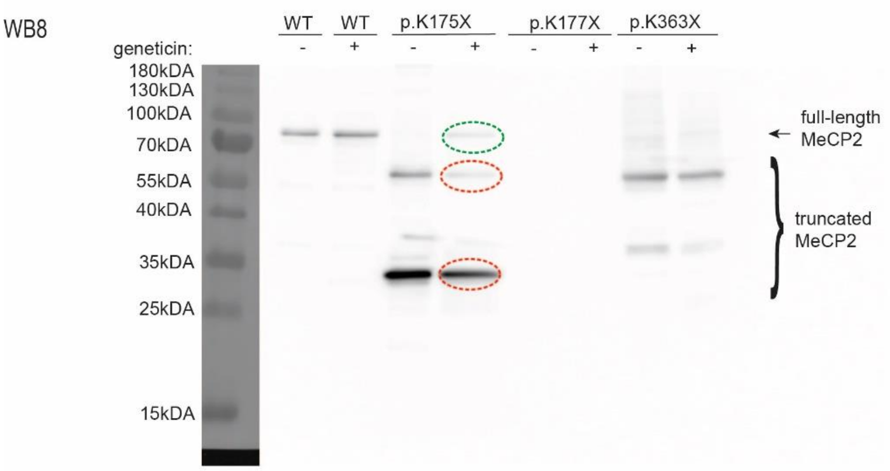

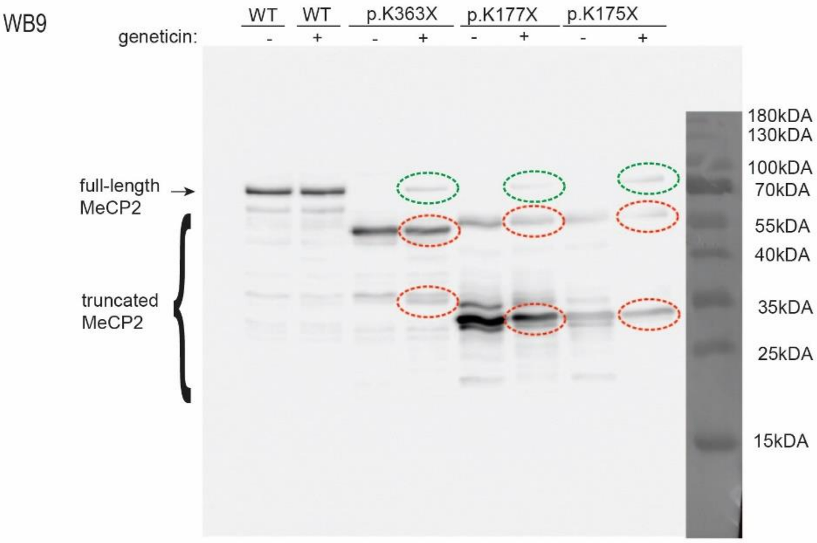

used in supplementary figure 1

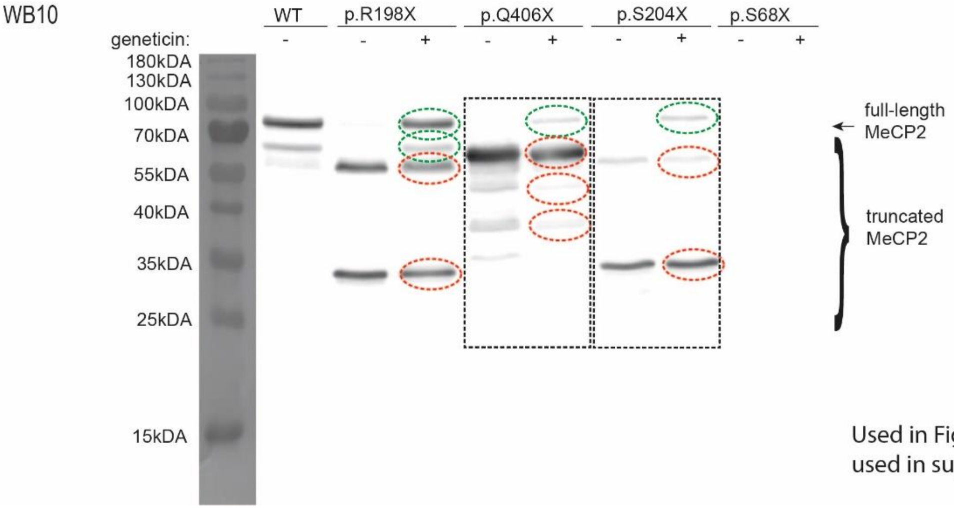

Used in Figure 4
used in supplementary figure 1

Readthrough band
Truncated band
Figure 4 Crop

WB11

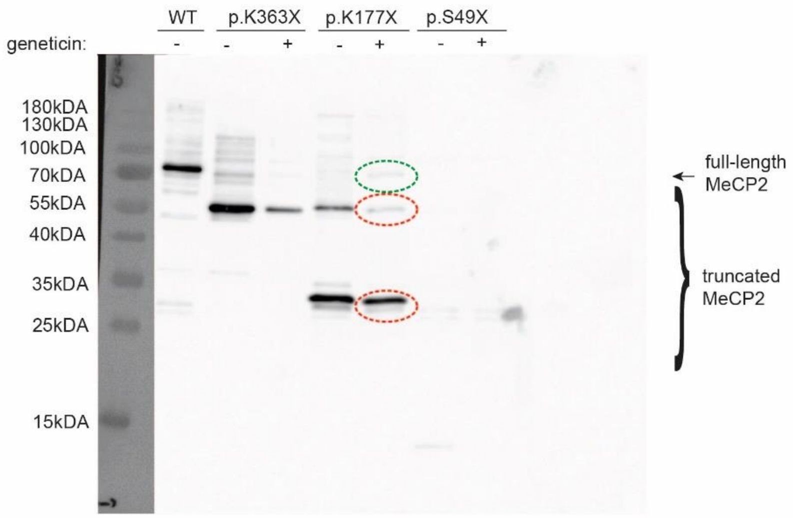

WB12

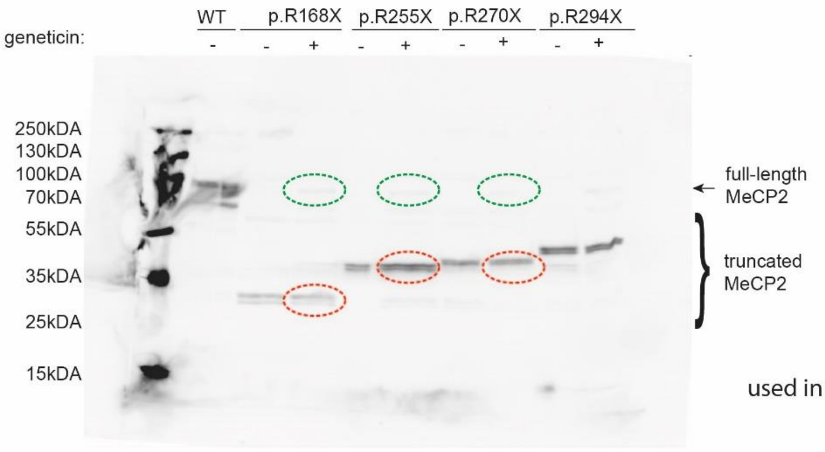

used in supplementary figure 1

WB13

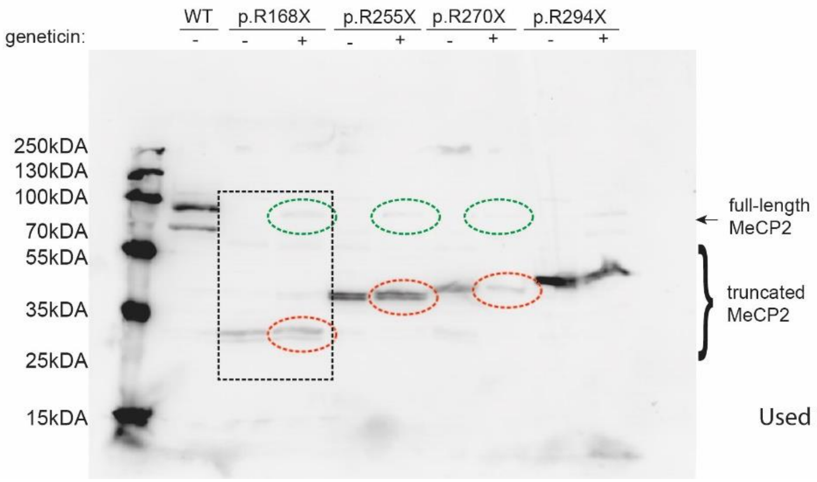

Used in Figure 4

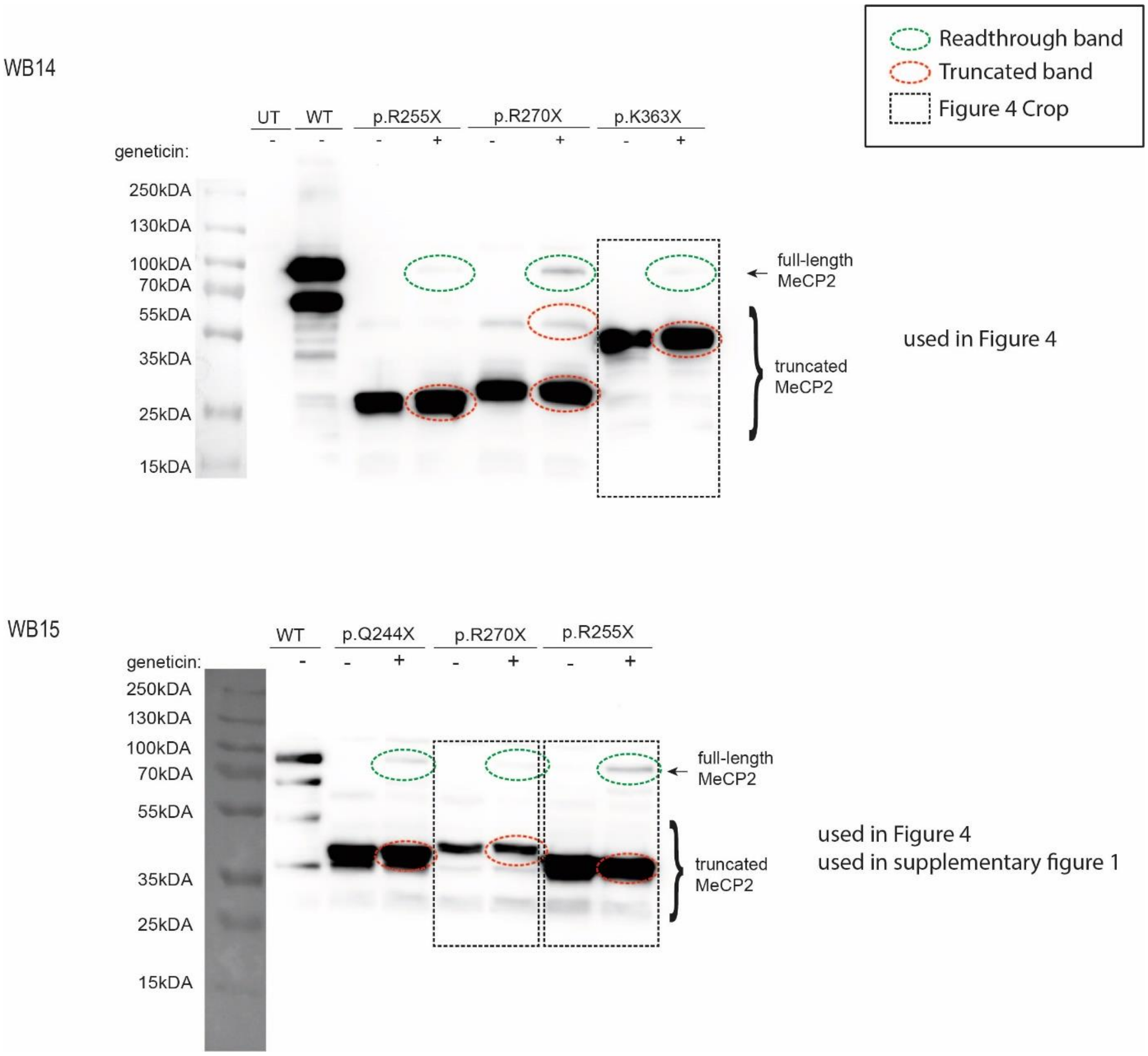
Readthrough band
Truncated band
Figure 4 Crop
WB14
UT
WT
p.R255X
p.R270X
p.K363X
geneticin:
- - - + - + - +
250kDA
130kDA
100kDA
70kDA
55kDA
35kDA
25kDA
15kDA
full-length MeCP2
truncated MeCP2
used in Figure 4
WB15
WT
p.Q244X
p.R270X
p.R255X
geneticin:
- - + - + - +
250kDA
130kDA
100kDA
70kDA
55kDA
35kDA
25kDA
15kDA
full-length MeCP2
truncated MeCP2
used in Figure 4
used in supplementary figure 1

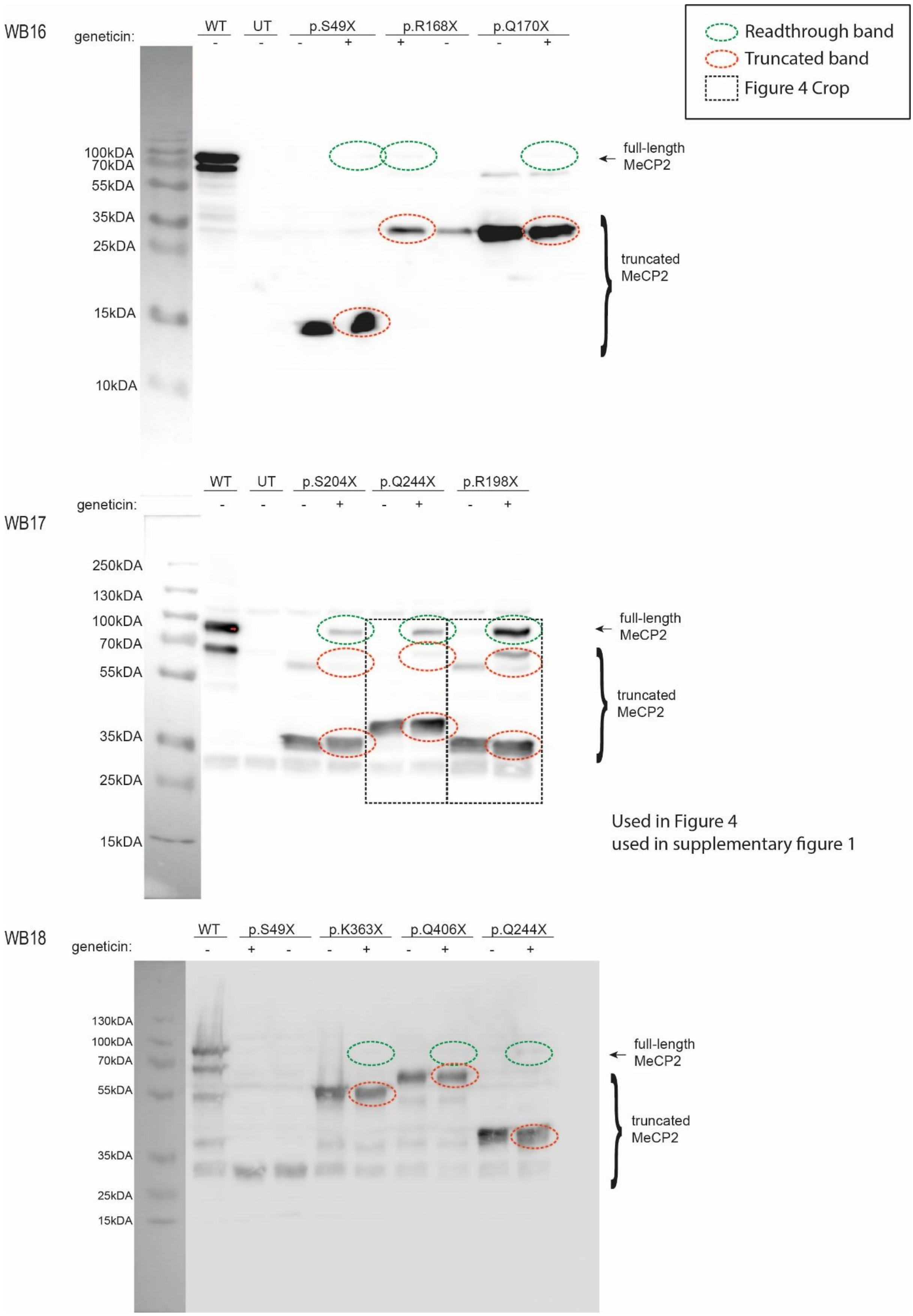
Readthrough band
Truncated band
Figure 4 Crop
WB16
geneticin:
WT
UT
p.S49X
p.R168X
p.Q170X
100kDA
70kDA
55kDA
35kDA
25kDA
15kDA
10kDA
full-length MeCP2
truncated MeCP2
WB17
geneticin:
WT
UT
p.S204X
p.Q244X
p.R198X
250kDA
130kDA
100kDA
70kDA
55kDA
35kDA
25kDA
15kDA
full-length MeCP2
truncated MeCP2
Used in Figure 4
used in supplementary figure 1
WB18
geneticin:
WT
p.S49X
p.K363X
p.Q406X
p.Q244X
130kDA
100kDA
70kDA
55kDA
35kDA
25kDA
15kDA
full-length MeCP2
truncated MeCP2

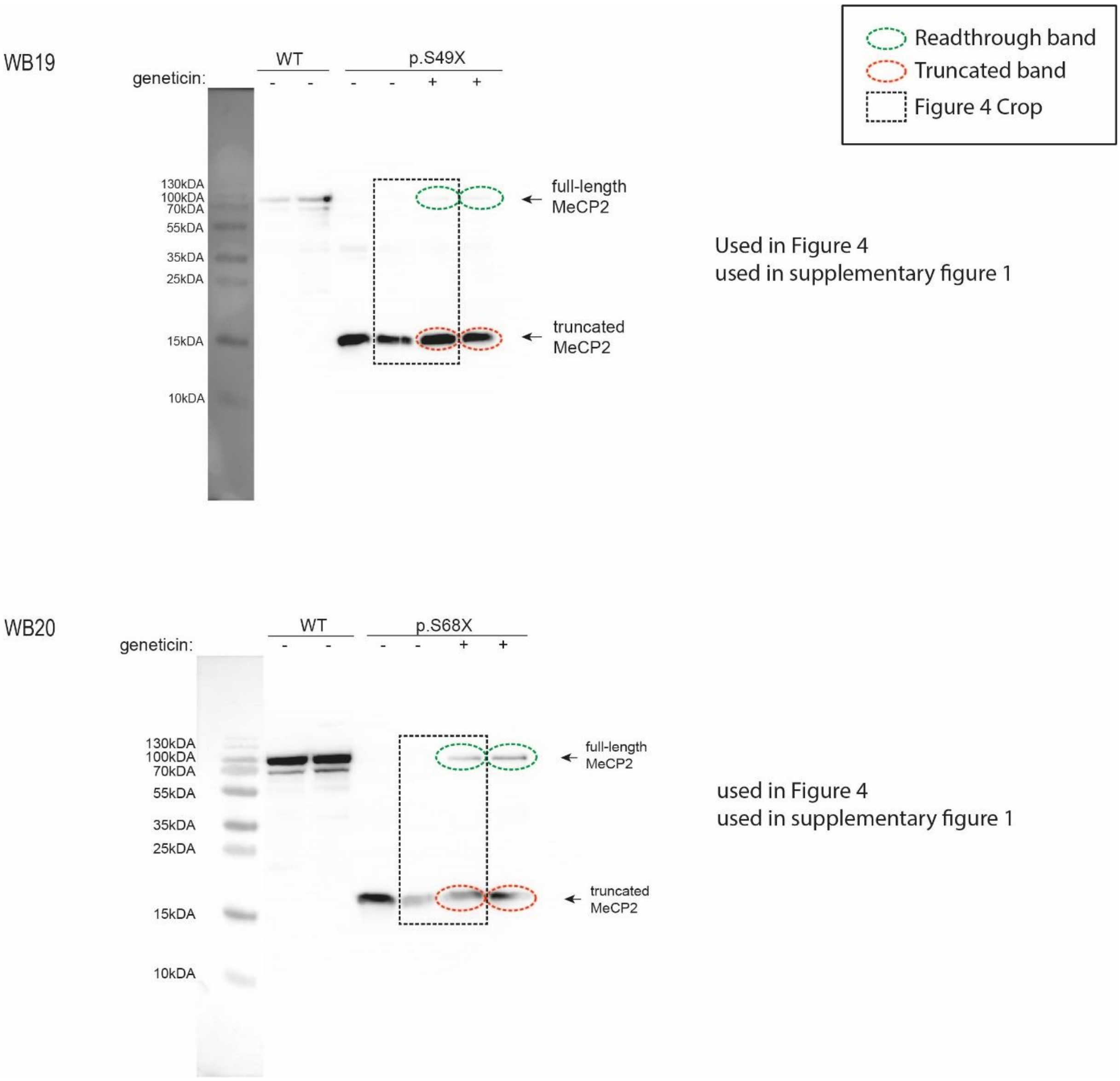

Supplementary Figure 3: Full unedited Western Blots (WB) 1-20 for Figure 4 and Supplementary Figure 2. Cropped areas for Figure 4 are marked in black dotted squares. All used bands for densitometric analyses are encircled in green for readthrough bands and red for truncated bands. Readthrough quantification was determined as the readthrough band intensity (green) normalized on the readthrough plus truncated band intensity.

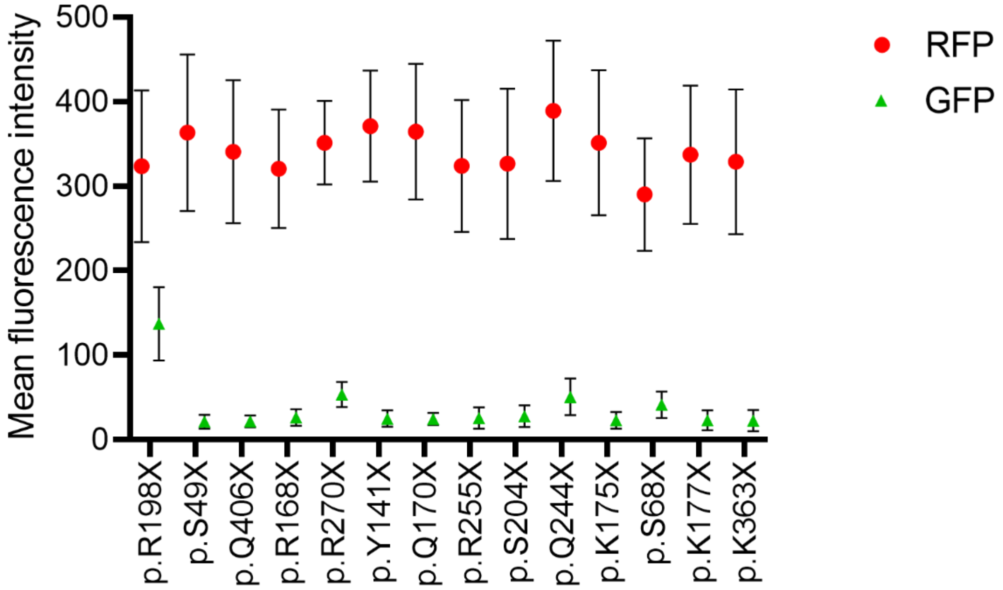

Supplementary Figure 4: Mean of all absolute fluorescence intensities of all constructs measured by the dual reporter system. A non-significant difference between all RFP intensities indicates stable expression of all reporter constructs. (n = 9, n = 6 for Q244X). Dots indicate mean, error bars indicate s.d. One-way ANOVA
